# Current Status on Stem Cells and Cancers of the Gastric Epithelium

**DOI:** 10.3390/ijms160819153

**Published:** 2015-08-14

**Authors:** Werner Hoffmann

**Affiliations:** Institute of Molecular Biology and Medicinal Chemistry, Otto-von-Guericke-University Magdeburg, Leipziger Str. 44, D-39120 Magdeburg, Germany; E-Mail: werner.hoffmann@med.ovgu.de

**Keywords:** gastric cancer, *Helicobacter pylori*, gastric mucosa, gastric self-renewal, cancer stem cell, gastric stem cell, metaplasia, SPEM, cell differentiation

## Abstract

Gastric cancer is still a leading cause of cancer-related mortality worldwide in spite of declining incidence. Gastric cancers are, essentially, adenocarcinomas and one of the strongest risk factors is still infection with *Helicobacter pylori*. Within the last years, it became clear that gastric self-renewal and carcinogenesis are intimately linked, particularly during chronic inflammatory conditions. Generally, gastric cancer is now regarded as a disease resulting from dysregulated differentiation of stem and progenitor cells, mainly due to an inflammatory environment. However, the situation in the stomach is rather complex, consisting of two types of gastric units which show bidirectional self-renewal from an unexpectedly large variety of progenitor/stem cell populations. As in many other tumors, cancer stem cells have also been characterized for gastric cancer. This review focuses on the various gastric epithelial stem cells, how they contribute to self-renewal and which routes are known to gastric adenocarcinomas, including their stem cells.

## 1. Introduction

Some 90% of fatal malignancies in adult humans arise from epithelia because of their high regenerative capacity, which is due to the existence of somatic stem cells. Amongst these, gastric cancer (GC) is still a leading cause of cancer-related mortality worldwide (10% of total cancer deaths in 2008,* i.e.*, 740,000 persons); for example, GC is the third leading cause for men and the fifth leading cause for women of global cancer mortality [1]. Within the last decade, both incidence and mortality have been declining substantially, particularly in developed countries. For example, GC is responsible for 3.7% (male) and 3.0% (female) of cancer-related deaths in Germany in 2008 (www.krebsdaten.de), respectively. Due to the development of diagnostic and therapeutic strategies there is excellent survival for patients with early GC. In contrast, the prognosis for patients at an advanced cancer stage is not very favorable. Generally, the five-year survival rate is <30% in most countries because of a rather late diagnosis for the majority of patients [2]. Thus, tools to enable early diagnosis are of critical importance [2].

Among GC patients, 90% develop adenocarcinomas; whereas 10% develop lymphomas and stromal tumors. Adenocarcinomas were classified histologically into the “intestinal” (50%, differentiated), the “diffuse” (33%, poorly differentiated), and the mixed or unclassified types (17%) [3,4,5]. Furthermore, a new classification has been recommended by the World Health Organization (WHO) in 2010 and, recently, a molecular characterization of gastric adenocarcinoma has been proposed [6]. The latter classification defines four major subtypes (chromosomal instability/intestinal histology, genomically-stable/diffuse histology, microsatellite unstable, and Epstein–Barr virus-positive tumors), which also show different distributions in the distinct regions of the stomach [6].

GC is promoted by environmental factors (such as food high in salt, smoked or cured meat/nitrates/nitrites, chili peppers, alcohol, smoking, as well as a diet low in fruits and vegetables, and low fiber intake) [7]. However, the most striking risk factor for GC is infection with *Helicobacter pylori*, which was discovered in 1984 by Marshall and Warren and classified since 1994 by the WHO as a class I carcinogen [8,9]. This concept has been proven by a prospective study in 2001, which clearly demonstrated that only patients with *H. pylori* infection developed GC [10]. Infection with Epstein–Barr virus is another environmental risk factor for GC. Furthermore, there are also well-known genetic risk factors for GC, such as sex (higher risk for males), hereditary diffuse GC (mutation in E-cadherin/*CDH1*), gastric adenocarcinoma and proximal polyposis of the stomach (GAPPS), and other inherited cancer predisposition syndromes, such as familial adenomatous polyposis (FAP; mutations in *APC*), hereditary nonpolyposis colorectal cancer (HNPCC/Lynch syndrome; mutations in *hMLH1*, *hMSH2*), Li-Fraumeni syndrome (mutations in *P53*), Peutz-Jeghers syndrome (mutations in *STK11*), and *BRCA2* mutations [7,11].

For the intestinal type of gastric adenocarcinomas, a well-defined sequence of events was described in 1988 on the base of chronic inflammation, known as the “Correa cascade”, starting from chronic gastritis and proceeding to gastric atrophy (*i.e*., a loss of parietal and zymogenic cells), intestinal metaplasia (IM), and dysplasia. Only in 1992 was this model extended by introducing *H. pylori* as a major causative agent of chronic inflammation and GC [8,12,13,14]. In contrast, the diffuse type of gastric adenocarcinomas, which can also result from *H. pylori* infection, arises in the absence of metaplastic changes [10].

Here, a brief overview is presented concerning the various gastric epithelial stem cells, how they contribute to continuous self-renewal of the gastric epithelium, and which routes are known leading to gastric adenocarcinomas including their cancer stem cells (CSCs).

## 2. Gastric Self-Renewal and Gastric Stem Cells

**Figure 1:**
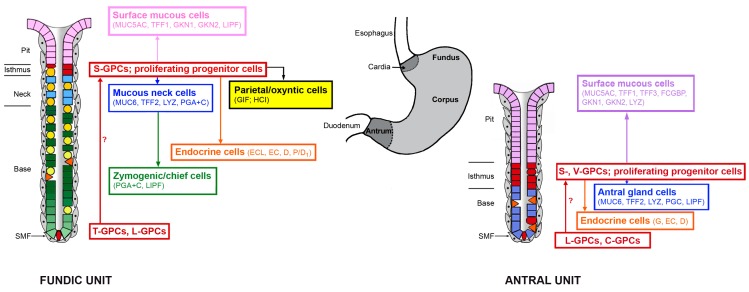
Schematic representation of the two gross types of human gastric units and their continual, bidirectional self-renewal from gastric progenitor cells (GPCs)/stem cells. Shown are the major epithelial cell types and some of their characteristic secretory products such as MUC, mucins; TFF peptides, FCGBP, IgG Fc binding protein; GKN, gastrokines; LYZ, lysozyme; LIPF, gastric lipase, PGA and PGC, pepsinogens; GIF, intrinsic factor; and HCl, hydrochloric acid; as observed in the classical fundic and antral units, respectively. The various gastric precursor cell (GPCs)/stem cell populations are marked in red: C, CCK2 receptor^+^; L, Lgr5^+^; S, Sox2^+^; T, Troy^+^; V, villin^+^; EC(L), enterochromaffin(-like); G, gastrin; SMF, subepithelial myofibroblast; (modified and updated from [15,17]).

The human gastric mucosa and its glands,* i.e.*, the gastric epithelium, differ histologically along the anterior-posterior (AP) axis and three zones can be distinguished: the cardiac zone, the fundus/corpus zone, and the antral/pyloric zone (Figure 1) [15]. The gastric mucosa contains about 3 million funnel-shaped pits (also called foveolae), which form the entrance into the gastric glands (divided into the isthmus, the neck, and the base). There are two gross types of gastric glands (Figure 1),* i.e.*, a fundic type (mainly in the fundus/corpus zone) and an antral type (in the cardiac and the antral/pyloric zones). Up to seven glands can open into the bottom of a single pit and new glands are generated by gland fission [16]. The combination of a pit and its associated glands is called a gastric unit [16]. Fundic units contain five principal mature epithelial cell types, which are of endodermal origin, whereas the simpler antral units are made of three predominant types of mature epithelial cells (Figure 1). A sheet of subepithelial myofibroblasts (SMF), which are of mesodermal origin, surrounds the gastric epithelium (Figure 1).

### 2.1. Bidirectional Self-Renewal of Gastric Units

**Figure 2:**
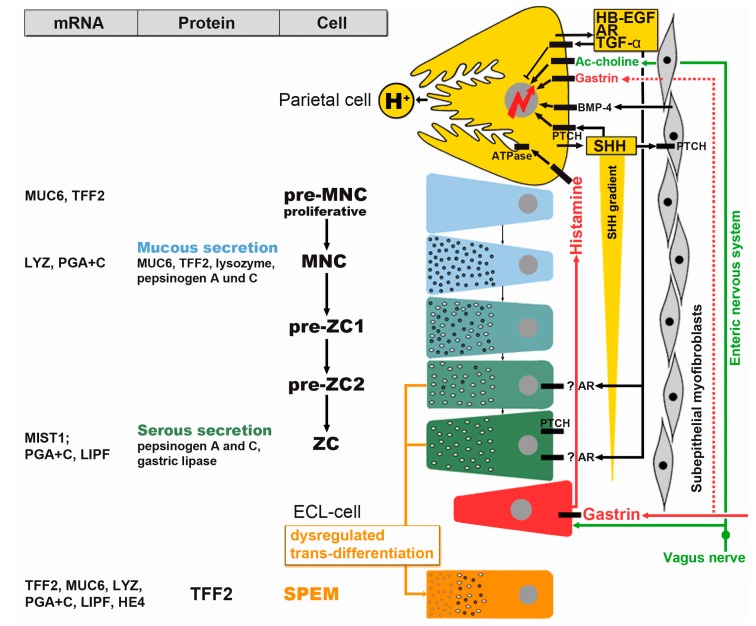
Schematic representation of the trans-differentiation of mucous neck cells (MNC, blue) towards zymogenic cells (ZC, green) under the control of parietal cells (yellow); the dysregulated trans-differentiation results in formation of the “spasmolytic polypeptide-expressing metaplasia” (SPEM; orange), which characteristically secretes TFF2. Immature progenitors for MNCs (pre-MNC) in the proliferative region of fundic units mature stepwise (pre-ZC1, pre-ZC2) towards ZCs, which is accompanied by a re-organization of the secretory apparatus (mucous to serous). Shown, also, is the distribution of characteristic transcripts and proteins, which is not congruent in these cells. AR, amphiregulin; BMP, bone morphogenetic protein, HB-EGF, heparin-binding EGF-like growth factor; PTCH, Patched; TGF-α, transforming growth factor α; (modified and updated from [17,27]).

In a series of elegant studies, Leblond and his co-workers identified the isthmus and neck regions as the major site of proliferation [18] and subsequently established the continual self-renewal of the murine gastric epithelium in both the corpus and the antrum, where cells differentiate from progenitor cells as they migrate bidirectionally toward the pit and the gland base, respectively (Figure 1) [15,19]. Particularly, the stepwise differentiation of the mucous neck cell (MNC)-zymogenic cell lineage in fundic units has received much attention, including the essential role of parietal cells as organizers of the fundic units due to their secretion of various epidermal growth factor (EGF) receptor ligands (such as transforming growth factor α, amphiregulin, and heparin-binding EGF-like growth factor), Sonic hedgehog (SHH), and HCl (Figure 2) [17,20,21,22,23]. The number and activity of parietal cells is pH-dependently regulated by gastrin, which is released into the blood stream from antral endocrine G cells (Figure 2). Gastrin is believed to act mainly indirectly by releasing histamine from fundic ECL cells, which in turn stimulates parietal cells in a paracrine fashion. Gastrin is considered only as a relatively poor direct stimulant of acid secretion from parietal cells. Enterochromaffin-like (ECL) cells and parietal cells are also innervated by the enteric nervous system (Figure 2). The morphogen SHH forms a gradient along the fundic gland axis allowing bidirectional communication with subepithelial myofibroblasts via Patched (PTCH) and bone morphogenetic protein (BMP)-4 (Figure 2). Of note, parietal cells form a niche for MNC-zymogenic progenitors in the neck [24]. Interestingly, MNC progenitors were characterized by their expression of TFF2 (previously termed spasmolytic polypeptide) transcripts (Figure 2), but not the corresponding protein, as shown for the mouse [25] and human [26].

Three immature cell types were identified in the isthmus of murine fundic units due to their high radioautographic labelling index: “granule free cells” and progenitors for surface mucous cells and MNCs, respectively [28]. Interestingly, the first report on various mitotic progenitor cells within the isthmus and neck regions of fundic glands responsible for a bidirectional regeneration mechanism was documented as early as 1893 [29]. Based on radioautographic (highest labeling index) and morphological data (lack of secretory granules), the undifferentiated “granule-free cells” in the isthmus of murine antral and fundic units were suggested to represent gastric epithelial stem cells [28,30]. Surprisingly, no direct morphological counterpart was detected in human fundic units. The least differentiated immature cells described in the mid isthmus were rare “mini-granule cells” [31]. These cells contain granules with varying density and appearance but fairly uniform size (diameter: 210 ± 15 nm) and it is questionable if they represent stem cells [31].

For a long time, and in contrast to the recent picture (Figure 1), the isthmus of both the fundus and the antrum, with its proliferating cells, was generally believed also to exclusively harbor the adult (somatic) stem cells. These undifferentiated cells, often located within a niche, are defined by two characteristics [8,15]. First, they have the ability to maintain themselves, long-term, by symmetric cell division (self-renewal). Second, adult stem cells are the origin of all the cell-lineages of a respective tissue/gland due to asymmetric cell divisions leading also to various types of progenitor cells, which proliferate (transit-amplifying cells) and finally differentiate into the mature cell types (multipotency). Indeed, expression profiling from laser capture microdissected adult mouse corpus progenitor cells from this zone revealed similarities with other stem cell populations.  Furthermore, Wnt/β-catenin, PI3K/Akt, transforming growth factor-β (TGF-β), and insulin-like growth factor-1 signaling cascades were recognized as the top scoring networks and there were also indications that these cells represent a complex interface with the nervous system and the microvasculature [32]. Of note, the transgenic mice investigated here had their parietal cells ablated, which could alter the normal expression profile. Furthermore, Doublecortin and CaM-like kinase 1 (Dclk1/Dcamkl1) was expressed in pre-MNC progenitors in the isthmus and thus was suggested as a first marker of adult gastric stem cells [32]. However, a similar cell population has been characterized by its Tff2 expression which gives rise to MNCs, parietal and chief cells only [25]. Thus, it is questionable if Dclk1 is a marker for stem cells in the isthmus. Remarkably, Notch signaling is also largely confined to the proliferative zone in the murine isthmus and Notch activation is sufficient to induce dedifferentiation of lineage-committed epithelial cells into multipotential progenitors indicating an astonishing plasticity of these cells [33].

Defining stem cells is not a trivial task. Nowadays, mainly two experimental methods are used, *i.e*., lineage tracing studies using transgenic animals and the generation of organoids *in vitro*.

### 2.2. Gastric Units Contain Multiple Progenitor/Stem Cell Populations

A first* in vivo* lineage tracing study in the adult mouse clearly demonstrated that both fundic and antral units contain multipotential stem cells capable of generating all epithelial cell types [34]. All epithelial cells in an individual gland appear to be derived from a single stem cell and the clonal expansion occurred more rapidly in the antrum than in the corpus [34]. Furthermore, parietal and zymogenic cells seem to have lower turnover rates than the other cell lineages. An important clonal tracing study in the human stomach clearly showed that there are multiple stem cells present in a single gastric unit, but each individual gland seems to be populated by descendants of a single stem cell [16]. Furthermore, a single stem cell can also expand and colonize the entire unit, a process called monoclonal conversion [16]. However, the identity of the stem cells was not revealed in either of these studies.

A major breakthrough was the use of genetic markers and* in vivo* lineage tracing for the identification of multipotential gastric progenitor cells (GPCs)/stem cells in the murine stomach [35]. In 2007, this approach first allowed the identification of a rare population of cells predominantly in the lesser curvature of antral units of the mouse at or below the isthmus on their basis of villin transgene expression (V-GPCs) [36]. Only about 200–400 V-GPCs are present in the adult mouse stomach,* i.e.*, in average less than one cell per antral gland [36]. These cells are normally quiescent, but multiply in response to the pro-inflammatory cytokine interferon-γ where they give rise to all lineages of antral glands [36]. V-GPCs do not contribute to self-renewal under normal conditions, are nearly absent in fundic units, and probably serve as a reserve stem cell pool that is activated in response to damage or inflammation.

In 2010, another population of GPCs was identified in the mouse, which characteristically express the stem cell marker Lgr5 (L-GPCs), particularly at the base of antral units in both the antrum and cardia [37], but obviously also at the base of fundic units [38,39]. Also in humans, LGR5 expression was demonstrated at the base of antral glands but not in fundic units [40]. Antral L-GPCs proliferate (a portion between 10% [41] and 29% [37] were reported to be actively cycling) and lineage tracing demonstrated that they function as multipotent, self-renewing stem cells in antral units; they can even build long-lived gastric organoids* in vitro* [37]. About eight L-GPCs are active in each gland base [42] and a single cell can achieve clonal dominance. However, the precise relation between the L-GPCs at the gland base and the progenitor cells in the isthmus is currently not known and a rapid migration of the immediate L-GPCs progeny up to the isthmus and further amplification is reasonable. Lgr5, a 7-transmembrane receptor binding R-spondin as a ligand [43], is a Wnt target gene and multiple additional Wnt target genes were also selectively expressed in L-GPCs indicating robust Wnt signaling in these cells [37]. However, the source of the Wnt ligands has not yet been established. Possible sources include neighboring apoptotic antral gland cells and subepithelial myofibroblasts [44]. Furthermore, innervating nerves can activate Wnt signaling in gastric stem cells through the muscarinic acetylcholine M_3_ receptor [45] and certain stem cells also can propagate even in an autocrine fashion [44].

Recently, an additional stem cell population has been identified about at position +4 in murine antral glands, which is characterized by expression of the gastrin CCK2 receptor [41]. These C-GPCs are localized slightly above typical L-GPCs and treatment with progastrin, but not amidated gastrin, interconverted C-GPCs into L-GPCs; furthermore, increased gastric stem cell number and gland fission was observed and* in vitro* cultures of C-GPCs robustly formed gastric organoids [41]. Thus, C-GPCs represent antral stem cells which can be interconverted by a hormonal trigger.

A further population of stem and progenitor cells was discovered in the murine stomach in 2011, which has been characterized by their expression of the stem cell marker Sox2 (S-GPCs) [46]. These cells are scattered throughout the isthmus in both the fundic and antral units as well as in lower parts of the glands and they give rise to all mature gastric cell types [46]. Furthermore, S-GPCs have the potential to self-renewal and they did not show an apparent overlap with the population of L-GPCs [46]. The expansion in antral units occurred more rapidly than in fundic units, which is in agreement with the slower turnover rate of the fundic units. Interestingly, S-GPCs originate from fetal Sox2^+^ tissue progenitors [46]. Taken together, S-GPCs might be the cells with the closest relation to the gastric stem cells originally suggested in the proliferative zone of the isthmus [19]. However, ablation of Sox2^+^ cells did not result in an obvious phenotype in the glandular stomach [46].

In completing the situation in fundic units, a small subpopulation of dividing and fully-differentiated zymogenic cells has been identified in 2013 at the base of murine fundic units, which surprisingly express the intestinal stem cell marker *Troy*^+^ (T-GPCs) [38]. T-GPCs comprised about 0.9% of all epithelial corpus gland cells in the mouse. These cells were able to act as multipotent stem cells of the entire fundic unit over periods of months and this phenomenon was accelerated upon tissue damage; furthermore, T-GPCs can be cultured to generate long-lived gastric organoids [38]. Thus, T-GPCs are thought to act as quiescent “reserve” stem cells [38]. Surprisingly, T-GPCs express Wnt target genes, but the source of the Wnt ligands at the gland base is not known thus far [38]. Furthermore, also the connection of T-GPCs and the proliferative region at the isthmus has not yet been established. The SHH gradient along the fundic gland axis [20,22] may serve as a guide towards the isthmus.

Taken together, both the fundic and the antral units contain various populations of GPCs/stem cells (*i.e*., T-, L- and S-* versus* L-, C-, S- and V-GPCs). In contrast to prior assumptions, the isthmus is not thought any more to serve as the exclusive stem cell reservoir. Stem cell populations/GPCs reside also at the bottom of the gastric units (T-, L-CPCs) and somewhat above (C-GPCs). On top, fundic and antral units differ markedly (Figure 1). Such a complex situation is not unusual because the coexistence of different stem cell populations is a common feature in mammals [47,48]. Stem cell populations can also differ in their proliferation rates, *i.e*., they can divide slowly (quiescent reserve population) or they are actively cycling cell populations [47]. Thus far, the relation and the hierarchy, respectively, between the different GPC/stem cell populations in a single gastric unit have not been established and, also, the actively dividing population(s) at the isthmus remains to be characterized. Furthermore, the question also arises as to how monoclonality of the gastric units is maintained in spite of multiple stem cell populations. One explanation would be a competition model where a single stem cell can ultimately achieve clonal dominance [48]. Generally, the picture emerges that GPCs/stem cells show a higher degree of plasticity than expected by switching back and forth between committed and multipotential stages, maybe by the influence of Notch signaling [33]. Furthermore, the absolute requirement of niches is also seen less dogmatically now [44]. Thus far, most results were obtained from the murine stomach and it will be an important future goal to establish the distribution and the hierarchy of the various GPC/stem cell populations also in the human gastric epithelium.

A major step in finally reaching this goal was the generation of additional human gastric organoids *in vitro* from the corpus and the antrum, respectively [39,49]. For example, organoids from the corpus in the presence of Wnt and R-Spondin1 resemble glands whereas, without Wnt, organoids differentiate into pit lineage cultures [39]. This is a strong indication for a Wnt source at the bottom of these glands with a decreasing gradient towards the pit. Such an arrangement is perfectly designed to specifically regulate the fate of L-GPCs and T-GPCs at the gland bottom because Lgr5 and Troy are established Wnt target genes. This situation is also comparable with that in intestinal crypts where Paneth cells supply Wnt ligands [44].

### 2.3. Generation of the Anterior-Posterior Axis

The spatial organization of the gastric epithelium and its glands is tightly regulated and is characterized by four different axes.* i.e.*, the AP axis, the lateral (left-right) axis, the dorsal-ventral axis, and the radial (individual gland) axis [17]. Initial patterning of the endoderm and organogenesis occurs mainly via the AP axis during embryonic development; whereas the radial axis is the last to occur and which is maintained probably by a Wnt gradient [39]. These two axes are also the most characteristic ones and active patterning along these axes is maintained throughout life. The precision of these patterning events is essential for correct self-renewal and homeostasis of both the fundic and antral units. These units differ drastically along the AP axis in both their mature epithelial cell lineages and their multiple GPC/stem cell populations (Figure 1). Furthermore, surface mucous cells also differ along the AP axis; for example, they secrete the morphogen Indian hedgehog (IHH) with a steep increasing gradient from the fundus to the antrum [17]. Nowadays, the best markers defining the borderline between the gastric body and the antrum are ghrelin (body) and gastrin (antrum) [50]. Only recently, the *de novo* generation of human gastric organoids by directed differentiation of human pluripotent stem cells* in vitro* has been described which recapitulates normal embryonic development, particularly of the antrum [49]. Induction of an antral phenotype (including L-GPCs) from definitive endoderm cultures was accomplished by a combinatorial treatment with the growth factors/antagonists WNT3A, FGF4, noggin, retinoic acid and EGF [49]. It became clear that the fundus is defined by a combination of the transcription factors SOX2^+^/PDX1^−^; whereas the antrum is characterized by SOX2^+^/PDX1^+^ and the duodenum by PDX1^+^/CDX2^+^[49]. Of note, adult human antrum also contains individual units with pure fundic characteristics (parietal and chief cells) as well as a substantial portion of mixed units with both antral and fundic characteristics (but no chief cells) [50]. Of note, the variants with pure fundic characteristics in the antrum have fewer parietal cells per unit and a more prominent foveolar region when compared with classical fundic units [50]. However, PDX1 expression was restricted to the classical antral cell types, even in mixed glands, and not observed in parietal cells [50]. This is an indication that more than one type of GPC exists in antral units and the different lineages might originate from different GPCs.

## 3. Gastric Adenocarcinomas, Dysregulated Self-Renewal, and Gastric Cancer Stem Cells

Gastric cancers are, essentially, adenocarcinomas. The majority of GC is thought to originate on the base of chronic inflammation and in particular from *H. pylori* infection [10]. Most of these patients develop intestinal-type adenocarcinomas. According to a recent molecular classification, these are tumors with chromosomal instability [6]. A crucial step during intestinal-type carcinogenesis is the development of metaplasias [8,12,13,14]. As outlined in the following chapters, both *H. pylori* infection and metaplasias are tightly connected to dysregulated gastric self-renewal.

Furthermore, also hereditary forms of GC are intimately associated with dysregulated gastric self-renewal and gastric stem cells. For example, both humans (FAP) and mice with *APC* mutations, which affect Wnt signalling, develop cancers predominantly in the antrum establishing the L-GPCs as a potential cell of origin for gastric cancer [37,47].

Last, but not least, the different distribution of the molecular subtypes of adenocarcinomas in the distinct regions of the stomach [6], *i.e.*, the cardia, corpus, and antrum, is also a strong indication that the mode of self-renewal and the type of GPC/stem cell significantly determine the type of adenocarcinomas.

### 3.1. Consequences of H. pylori Infection

*H. pylori*-induced chronic gastritis is the strongest risk factor for non-cardia GC. This is in line with the importance of chronic inflammation as a general hallmark of carcinogenesis [51]. Predominant colonization of the gastric body by *H. pylori* leads to corpus-predominant inflammation, mucosal atrophy with a loss of parietal and chief cells, hypochlorhydria, and IM; whereas predominant *H. pylori* infection of the antrum causes hyperchlorhydria and predisposes to duodenal ulcer [9,14]. *H. pylori* infection results in characteristic proinflammatory signaling,* i.e.*, activation of the nuclear transcription factors NF-κB and AP-1 as well as release of interleukin-8 (IL-8) and tumor necrosis factor α (TNFα) [9,52]. This is even observed in human gastric organoids [39]. Of note, the immune response of the gastric mucosa has also been reported to be regulated by SHH from parietal cells because SHH is induced by *H. pylori* infection and acts as a macrophage chemoattractant [53]. Furthermore, a population of *H. pylori* is also able to penetrate and directly colonize the antral glands, leading to proliferation and expansion of L-GPCs, which alters turnover kinetics and the hyperplastic responses [54]. Interestingly, *H. pylori*-induced epithelial proliferation even in an antral gastric organoid [49]. This is in line with a report that *H. pylori* infection is associated with DNA damage of L-GPCs [55].

A hallmark of carcinomas (epithelial tumors) is a drastic change in their phenotype in order to become motile,* i.e.*, from apical-basolateral (in epithelial cells) towards planar polarity (mesenchymal phenotype) [56]. This characteristic change is known as the epithelial-mesenchymal transition (EMT) and is also associated with the acquisition of stem-like characteristics, such as expression of Bmi1 [57,58]. Thus, it is an important observation that *H. pylori* initiates EMT via the transcription factor ZEB1 and the microRNA miR-200 [59,60]. The classical marker for the epithelial phenoptype is E-cadherin/*CDH1*; thus, it is not astonishing that hereditary diffuse GC is associated with *CDH1* germline alterations [6,11].

### 3.2. Metaplasias

Within the last years it is has become clear that understanding the self-renewal of gastric units is a pre-requisite for understanding gastric carcinogenesis because dysregulated gastric self-renewal can lead to abnormal differentiation, where gastric epithelial cells are replaced by epithelial cells of another type (metaplasia). Metaplasias are the most documented process for GC [5]. Two such metaplastic lineages are well known in the stomach,* i.e.*, the IM and the “spasmolytic polypeptide-expressing metaplasia” (SPEM; characterized by its TFF2 expression) [61,62]. The latter is found at the base of fundic units and results from dysregulated trans-differentiation of zymogenic cells as well as on arrest of MNC trans-differentiation into zymogenic cells (Figure 2) [63,64]. Generally, SPEM develops in various settings after loss of the fundic organizers,* i.e.*, the parietal cells; the critical step is a loss of the essential secretory products of the parietal cells, such as SHH and various EGF receptor ligands [17,21,23,27,35,62]. Interestingly, also the loss of the disulfide isomerase AGR2, which is involved in the processing of the mucin MUC6 in MNCs, leads to SPEM [65]. Of special note, SPEM gives rise to IM and is even more strongly associated with GC than is IM [62]. Thus, it has been proposed that cancer cells arise from SPEM or from a proliferative intermediate generated during the further differentiation into IM [62]. At least for IM, a clear clonal evolution from metaplasia to dysplasia has been demonstrated in the human stomach and a single clone was able to expand to form an entire dysplastic lesion by field cancerization [66]. It would be interesting to show if such a relation exists also for SPEM and dysplasia, as proposed. Furthermore, the question concerning the origin of IM also in the antrum might be cleared with respect to the observation that individual units with pure fundic characteristics also occur in the antrum [50]. Particularly these units might be able to develop SPEM and subsequently IM in the antrum.

Alternatively, a second route has been described leading to intestinal-type adenocarcinomas in mice, where bone marrow-derived cells (BMDCs) were recruited to the stomach and repopulate the gastric mucosa only after chronic infection with different* Helicobacter* strains [8,13,67,68]. In contrast to *Helicobacter*-induced inflammation, which is an early event, BMDC repopulation of gastric glands is a late event, occurring after one year, and probably a subpopulation of bone marrow-derived mesenchymal stem cells (MSCs) adopt a gastric epithelial phenotype and are the source of gastric malignancy [8,67,68]. Of special note, acute injury, acute inflammation, or transient parietal cell loss do not lead to BMDC recruitment [67]. Mesenchymal stem cells utilize the CXCR4-SDF-1 axis for homing during acute, but not chronic, inflammation of the gastric mucosa [69]. Overexpression of SDF-1 in parietal cells induces dysplasia through expansion of stromal myofibroblasts and epithelial progenitors [70]. About 25% of the dysplastic lesions included cells that originated from the bone marrow [68]. This clearly indicates that the majority of dysplastic lesions do not originate from the bone marrow. However, engraftment of BMDCs from a donor into an organ has been clearly and unambiguously demonstrated to occur in humans, as well, and can even lead to adenocarcinoma [68,71].

### 3.3. Cancer Stem Cells

Nowadays, cancer is thought to derive from different “cells of origin” and tumors consist of a heterogeneous population of cancer cells which originate from a small subset of CSCs [72,73,74]. Only CSCs can initiate tumor formation, self-renew, differentiate into the various types of daughter cells, and metastasize (e.g., shown by their sphere-forming capacity* in vitro*, as well as tumorigenic abilities, in xenografts* in vivo*) [74,75,76]. This concept is the basis for the CSC theory [73,74] and has also been proven for GC [74,75,76]. In the past, tumors have been understood to originate from transformed adult stem cells [74]. CD44 was the first marker identified for gastric CSCs only in 2009 [77,78]. In addition, stem cell markers such as OCT-4, SOX2, CD133/Prom1, and NANOG have been recommended for identifying CSCs [75,79]. CD44 is a transmembrane cell-adhesion molecule, which has been used to identify CSC populations in other tumors as well. Various signaling pathways are discussed to control the self-renewal and differentiation of gastric CSCs, such as Hedghog, Wnt, Notch, various EGF receptor ligands, TGF-β/BMP, and NF-κB [76].

Currently, there are experimental data which suggest at least three different cellular origins of gastric CSCs. First, gastric CSCs are likely the result from dedifferentiation of metaplastic lineages (SPEM, IM) particularly in the corpus, which induced cancers in these regions [62,63,64,66]. Second, gastric CSCs can probably be generated from normal adult GPCs/stem cells [74] and the mutation can spread via field cancerization [16]. For example, (i) targeted deletion of Klf4 in V-GPCs induced transformation and tumorigenesis in the antrum of mice [80]; (ii) targeted disruption of Apc in L-GPCs resulted in the transformation of L-GPCs and adenomas were detected only in the antrum [37]; (iii) progastrin overexpression converted C-GPCs into L-GPCs followed by tumor initiation in the antrum [41]; (iv) all Tff1^KO^ mice spontaneously develop antropyloric adenoma and 30% progress to carcinoma [81]; treatment with a cyclooxygenase-2 (COX2) inhibitor,* i.e.*, a non-steroidal anti-inflammatory drug, suppressed tumor growth in the antrum of these mice [82]. One explanation would be that Tff1 specifically protects, e.g., L-GPCs from damage due to inflammation, thus acting indirectly as a tumor suppressor. Furthermore, this clearly indicates that the precancerous lesions in the antrum (adenoma) of Tff1^KO^ mice are linked to inflammation. Third, bone marrow-derived MSCs under the conditions of chronic inflammation have been demonstrated to be able to engraft into the gastric mucosa and lead to adenocarcinomas [67,68,71]. MSCs are commonly thought to be the most primitive uncommitted adult stem cells with particularly high multi-lineage potential [8,74]. Of note, their differentiation pattern mainly relies on the local microenvironment [74]. Such an intimate interaction has also been described for gastric CSCs [83]. MSCs are probably also more mutagenic than other cell types, they may transform more easily, and they particularly home to an inflammatory environment [8].

CSCs can be discriminated by their ability to efflux the fluorescent dye Hoechst 33342 (side population assay). Compared with their differentiated daughter cells, CSCs are also known for their increased resistance to chemo- and radiotherapies and often cause recurrence [76]. Thus, CSC-targeted therapies might be promising future strategies to cure GC [75,84]. Generally, gastric CSCs could be target of elimination or induced dedifferentiation, such as shown for therapy of acute promyelocytic leukemia with retinoic acid [84]. Potential targets are the transcription factors TR3 [79] and ATOH1 [85] as well as the ligands B7-H1 [86] and SHH [87].

## 4. Conclusions and Future Perspectives

Self-renewal and carcinogenesis are closely linked phenomena, particularly during chronic inflammatory conditions. Thus, GC includes a fairly wide spectrum of pathological processes mainly caused by dysregulated differentiation processes affecting normal stem cell populations, as well as metaplastic lineages. Thus, understanding the molecular details of self-renewal, generally, and also embryological development, will be of great advantage to unravel the processes leading to carcinogenesis. For example, it will be an important goal to establish the hierarchy of GPCs/stem cells particularly in humans, and also to clarify if there are additional stem cell populations in the isthmus not identified thus far. Additionally, the influence of the subepithelial myofibroblasts and their plasticity [17] is not completely understood. Major breakthroughs can also be expected from the generation and study of various human organoids [39,49]. Furthermore, the characterization of gastric stem cells will also be helpful for the understanding of cancers in regions adjacent to the stomach. For example, L-GPCs in the cardia are activated in Barrett esophagus [88]. For the clinic, in addition to developing specific strategies for preventing chronic inflammatory conditions, CSC-targeted therapies will probably be at the center of major developments.
